# Long-Term Impact of Chemical and Alternative Fungicides Applied to Grapevine *cv* Nebbiolo on Berry Transcriptome

**DOI:** 10.3390/ijms21176067

**Published:** 2020-08-23

**Authors:** Raffaella Balestrini, Stefano Ghignone, Gabriela Quiroga, Valentina Fiorilli, Irene Romano, Giorgio Gambino

**Affiliations:** 1 National Research Council, Institute for Sustainable Plant Protection, 10125 Turin, Italy; stefano.ghignone@ipsp.cnr.it (S.G.); gaquirogagar@gmail.com (G.Q.); irenedonatella.romano@uniroma1.it (I.R.); giorgio.gambino@ipsp.cnr.it (G.G.); 2 Department of Life Science and Systems Biology, Turin University, 10125 Turin, Italy; valentina.fiorilli@unito.it

**Keywords:** viticulture, berry transcriptome, laser microdissection

## Abstract

Viticulture is one of the horticultural systems in which antifungal treatments can be extremely frequent, with substantial economic and environmental costs. New products, such as biofungicides, resistance inducers and biostimulants, may represent alternative crop protection strategies respectful of the environmental sustainability and food safety. Here, the main purpose was to evaluate the systemic molecular modifications induced by biocontrol products as laminarin, resistance inducers (i.e., fosetyl-Al and potassium phosphonate), electrolyzed water and a standard chemical fungicide (i.e., metiram), on the transcriptomic profile of ‘Nebbiolo’ grape berries at harvest. In addition to a validation of the sequencing data through real-time polymerase chain reaction (PCR), for the first-time the expression of some candidate genes in different cell-types of berry skin (i.e., epidermal and hypodermal layers) was evaluated using the laser microdissection approach. Results showed that several considered antifungal treatments do not strongly affect the berry transcriptome profile at the end of season. Although some treatments do not activate long lasting molecular defense priming features in berry, some compounds appear to be more active in long-term responses. In addition, genes differentially expressed in the two-cell type populations forming the berry skin were found, suggesting a different function for the two-cell type populations.

## 1. Introduction

Grapevine is one of the most important cultivated fruit crops worldwide, whose fruits are consumed fresh or dried as table grapes or used to produce beverage (e.g., juice as well as wines and spirits after fermentation) and nutraceutical products. The processes involved in fruit development was intensively studied, unveiling the mechanisms correlated to ripening, to improve quality of production and, more recently, to maintain high quality under changing climatic conditions [1,2,3,4,5,6,7,8,9,10]. Most grown grapevine varieties (*Vitis vinifera cvs*) are susceptible to fungal/oomycetes-associated diseases, such as powdery and downy mildew, caused respectively by *Uncinula necator* (sin. *Erysiphe necator*) and *Plasmopara viticola*, leading to great yield losses and requiring numerous fungicide treatments [11]. Grapevine is one of the horticultural crops for which antifungal treatments can be extremely frequent, determining substantial costs at the economic and environmental level [12]. Besides the cost issue, the effects on the human health and the environment due to an overuse of pesticides should be considered [13]. The rising concern for a more sustainable vineyard management is among the reasons of the development of safe alternatives to conventional disease control methods [14]. Growing grape varieties resistant to mildews is one solution for reducing reliance on fungicides, although it is not enough by itself [15]. There is the need to identify new molecules/formulates or to improve the use of those already available, in a context of a more environmentally friendly viticulture. The effectiveness of these products, such as biofungicides, resistance inducers and biostimulants, applied alone or included in protection approaches in combination with routinely used fungicides, may lead to the development of alternative crop protection strategies respectful of the environmental sustainability and food safety [16]. Grapevine represents a model for studying interactions among plant, elicitors and pathogens [17,18]. Despite the growing interest in the use of these alternative products, few studies were conducted for the development and application of these solutions [19,20,21,22]. Additional efforts should be done to assess the effects of these strategies on the production and quality of grapes and wine, providing information useful for the companies acting in this sector. Recently, the efficacy of environmentally friendly products against downy and powdery mildews have been demonstrated in a vineyard of ‘Nebbiolo,’ a grape variety commonly used for production of renowned Barolo, Barbaresco and Roero PDO wines [22]. ‘Nebbiolo,’ as well as all varieties of *V. vinifera*, is very sensitive to attacks by fungi and oomycota in field conditions. Furthermore, it is particularly sensitive to powdery mildew more than other genotypes [18]. Rantsiou et al. [22] has already verified the impact of these compounds (e.g., laminarin and chito-oligosaccharides) on yield and vine vigor, skin features (including phenolic composition) and yeast community present on grape berries at harvest. Particularly, laminarin, which is extracted from the brown algae *Laminaria digitate*, is already known to act inducing resistance against downy and powdery mildew and grey mould in grapevine [23,24,25,26,27]. At molecular level, although berry transcriptomic profiles have been already studied with the aim to identify the candidate genes associated with phenotypic plasticity [6] and to developmental processes such as ripening [28] also in ‘Nebbiolo’ grape [10], few information are present on the changes associated to the application of biocontrol products. Here, the main purpose was to study the effect of a biocontrol product (i.e., laminarin), resistance inducers (i.e., fosetyl-Al and potassium phosphonate), electrolyzed water and metiram on the transcriptome of ‘Nebbiolo’ grape berries at the end of the season. Metiram has been chosen as the chemical reference product since, in the coming years, there will be a need to reduce its use and it is therefore necessary to deepen the studies on other alternative compounds that can support the effect of copper. In detail, we focused on the molecular modifications induced by the different treatments at the end of season, some weeks after their last application in vineyards. Furthermore, the expression of some candidate genes in different cell-types of berry skin was evaluated using the laser microdissection approach.

## 2. Results

### 2.1. Transcriptional Modifications Induced by Antifungal Treatments in Berry

Five commercial antifungal compounds, i.e., fosetyl-Al (Fos-Al), potassium phosphonate (K-Pho), laminarin (Lam), electrolyzed water (EOW) and metiram (Met), were applied in 2017 in a vineyard of ‘Nebbiolo’ following the scheme reported in Table S1 and previously described [22]. The antifungal compounds were used in combination with sulphur and copper since the experiment was conducted in a commercial vineyard and it was necessary to follow the legal rules for antifungal treatments. Therefore, the results obtained refer to the whole treatment used in the vineyard (Table S1) rather than to the single antifungal compound. 58% of clusters in the untreated control (CTRL) were affected by *U. necator*, all other treatments reduced the levels of powdery mildew infection and no symptoms associated to downy mildew were observed [22]. Lam and K-Pho were among the treatments showing the best control of the pathogen, followed by Fos-Al and Met, while EOW induced a very limited control of powdery mildew [22,27]. In the vineyard where the experiments were conducted, the vintage 2017 was characterized by low rainfall (Figure S1), especially in the summer months (July and August), thus inducing limited development of oomycete-associated diseases. However, this did not affect the evaluation of the effects of the alternative fungicides on long-term impact of on berry transcriptome. The molecular effect of the antifungal treatments on grape berries at harvest was firstly evaluated by RNA-seq analysis. Sequencing of RNA samples produced on average ~68 millions of raw reads per sample, reduced to 62 million after pre-processing, with a read loss ranging from 7.3 to 8.9 percent (Table S2). Processed reads ranging from 44.5 to 84.7 million for each sample were mapped onto the *V. vinifera* genome obtaining on average ~92.25% overall alignment rate, reaching an average gene coverage of 9.9X (Table S2). A total of 25,531 out of 30,609 genes have counts > 1 (Tables S3 and S4). Although a variability among the three biological replicates for each treatment exists (Figure S2), all the samples were considered in the subsequent analyses with the aim to exactly highlight the impact of the treatments in field.

**Figure 1:**
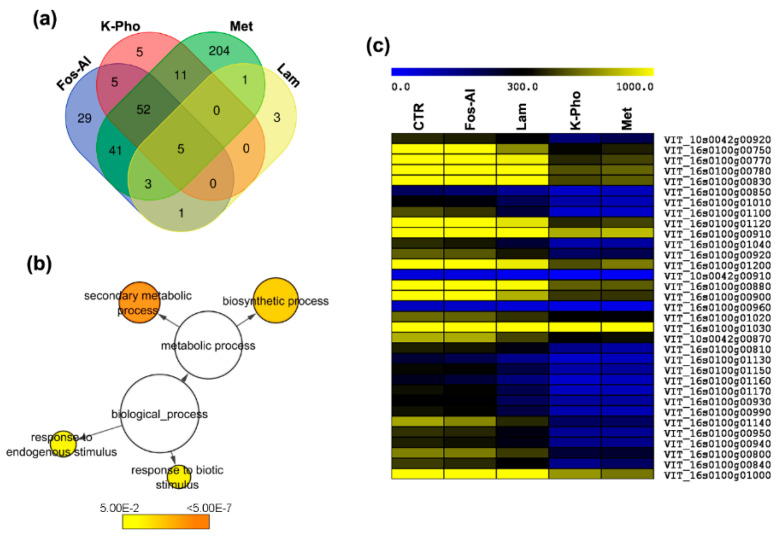
Overview of RNAseq data. (**a**) Venn diagram summarizing the differentially expressed genes in the several treatments (differentially expressed genes (DEGs)); (**b**) Enriched Gene Ontology (GO) terms. The network graphs show BiNGO (Biological Network Gene Ontology) visualizations of the overrepresented GO terms; (**c**) Hierarchical clustering analysis of transcripts corresponding to stilbene synthase gene family.

As a second step, to understand the systemic responses to treatments compared to untreated vines, transcriptional changes were determined by comparing Lam, Fos-Al, K-Pho, EOW and a standard chemical fungicide (Met) with untreated control (CTRL) sample. Only asymptomatic berries without symptoms of fungal attacks were collected for each treatment, including the samples collected from CTRL. Symptomatic berries were excluded from all the treatments because the aim of work was to explore the systemic effect of the treatments in asymptomatic grapevine tissues. In the EOW treatment, only three differentially expressed genes (DEGs) were observed with respect to CTRL and therefore EOW was excluded from subsequent analyzes. In total, 360 DEGs were found to be regulated in the four remaining treatments vs CTRL and, among them, 90 up-regulated and 270 down-regulated. Met treatment led to the regulation of a higher number of genes compared to the other treatments (Figure 1a).

Several DEGs presented were differentially regulated in unison among the treatments and specifically 5 DEGs are common among all the treatments (Tables S5 and S6, Figure 1a). Interestingly, among them, the only up-regulated is a gene coding for the 9-cis-epoxycarotenoid dioxygenase (VIT_19s0093g00550, previously reported as *VvNCED1* but renamed *VvNCED3* by Young et al. [29]), which is potentially involved in ABA biosynthesis. Looking at the DEGs specifically regulated in each treatment, 29 DEGs were found specifically in berries from Fos-Al treated plants, 3 DEGs in berries from Lam treated plants, 7 DEGs in berries after K-Pho treatment and 204 were specific in Met (Figure 1a). As opposed to Met treatment that led to the regulation of the highest number of genes among the treatments, Lam led to the regulation of very few genes, with only one up-regulated (*VvNCED3*) and 12 down-regulated. Three out of 13 DEGs were significantly downregulated specifically in this treatment, i.e., the genes coding for an early nodulin ENOD18 protein (VIT_04s0079g00610), a 1-aminocyclopropane-1-carboxylate oxidase 2 (VIT_11s0016g02380) with a role in ethylene biosynthesis and an unknown protein (VIT_03s0063g01760), respectively. Looking at the ten most upregulated genes among the several treatments (Fos-Al, K-Pho and Met), 6 transcripts were common among the conditions (Table 1 and Table S6). Particularly, in addition to *VvNCED3*, the three considered treatments led to a similar upregulation of genes coding for proteins with a role in different pathways such as genes coding for a putative aldehyde oxidase GLOX (VIT_02s0025g02600) that might have a role in defense, a putative omega-hydroxypalmitate O-feruloyl transferase (VIT_00s0207g00010) and putative wax synthase (VIT_12s0028g03480) potentially involved in cutin biosynthesis, a patellin protein (VIT_07s0031g03220) and a putative PIN (PINOID)-like protein (VIT_07s0031g02200). Conversely, a gene coding for a putative mannitol dehydrogenase is upregulated only in Fos-Al berries, while two genes (VIT_18s0001g08090 and VIT_19s0085g00920) coding respectively for an aux/IAA protein and an organic cation transport protein (OCT2) were specifically upregulated in K-Pho. To have an overview of the regulation of the main metabolic pathways involved in the different conditions, we conducted GO enrichment analysis. The most functional categories affected by treatments are biosynthetic and secondary metabolic processes and the responses to biotic and endogenous stimulus (Figure 1b, Table S7). It is worth noting the downregulation of all the members in response to biotic stress and secondary metabolism with respect to the infected CTRL and, among them, all the stilbene synthase genes (Figure 1c). Cell wall components have also an importance in plant defense as well as in grapevine berry developmental processes [7,8,10].

Here, only some cell wall genes appeared regulated in one or more treatments, that is, a cellulase gene (VIT_01s0137g00430) was downregulated in K-Pho and Met treated berries as well as a cellulose synthase gene (VIT_02s0025g01780) but only in Met. By contrast, a gene (VIT_12s0057g01020) coding for a putative fasciclin-like arabinogalactan protein (*VvFLA2*) was upregulated but only after Met treatment together with a gene (*VvXET*) coding for a xyloglucan endotransglucosylase (VIT_01s0150g00460) that resulted to be upregulated also in Fos-Al treated plants. This gene was previously found as suppressed just after véraison, while was upregulated between mid-ripening and full ripening, suggesting a role during the last step of ripening [10,30]. Differently from genes putatively involved in cutin and suberin/lignin component biosynthesis, that is, a wax synthase (VIT_12s0028g03480) previously found up-regulated in ‘Nebbiolo’ at harvest [10] and partially during ripening in ‘Corvina’ [30] and a omega-hydroxypalmitate O-feruloyl transferase (VIT_00s0207g00010), four genes coding for enzymes involved in pectin remodeling (VIT_15s0048g00510, VIT_15s0048g00500, VIT_16s0022g00700, VIT_08s0007g08330) resulted to be all downregulated in Met, two in K-Pho and one in Fos-Al treated plants (Table S6).

**Figure 2:**
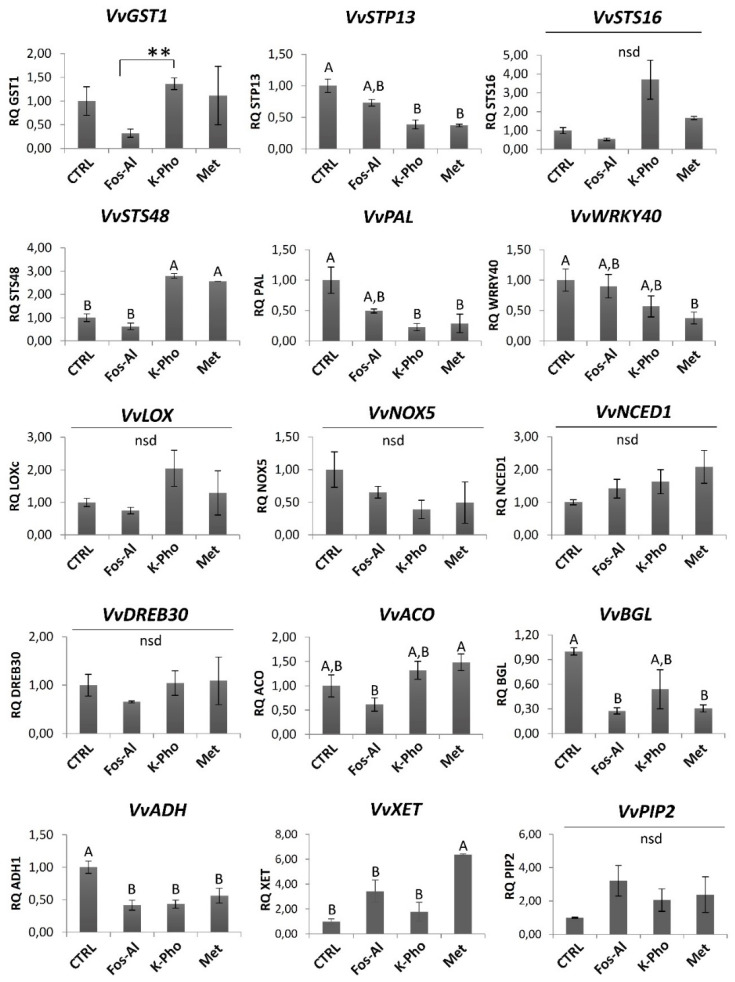
Relative gene expression of fifteen differentially expressed genes. RT-qPCR analysis was done on genes selected from DEGs identified in the comparison between the different treatments. The y-axis represents relative expression (RQ). Values are means of three replicates, while in Met treatment the mean value was obtained considering only two biological replicates for the listed genes: *VvGST1*, *VvSPT13*, *VvSTS16*, *VvLOXC*, *VvPIP2*, *VvXET*). Error bars represent standard deviation. Different letters denote significant differences (*p* ≤ 0.05) according to the one-way ANOVA with Tukey’s post-hoc test, while asterisks indicate significant differences (*p* ≤ 0.01) between data coming from Fos-Al and K-Pho (connection line).

RT-qPCR experiments were performed on independent RNA samples from CTRL, Fos-Al, K-Pho and Met treated plants with the aim to validate transcriptomics (Figure 2). Due to the low number of DEG, with respect to CTRL, in Lam treated plants, this treatment was not considered for the RT-qPCR validation. Fifteen regulated genes, representing a range of biological functions, were randomly chosen among the significantly regulated genes in the RNAseq experiment (Table S8). RT-qPCR data showed similar trends to RNAseq results for almost all of them (Figure 2), although differences between results obtained using the two techniques may reflect their different sensitivity but also the biological variation naturally occurred in field [22]. Results also allowed to highlight differences among treatments, for example, *VvXET* appeared significantly upregulated in Met in comparison with CTRL and the treatments and *VvACO* (VIT_11s0016g02380) that resulted in being downregulated (Figure 2).

### 2.2. Laser Microdissection

**Figure 3:**
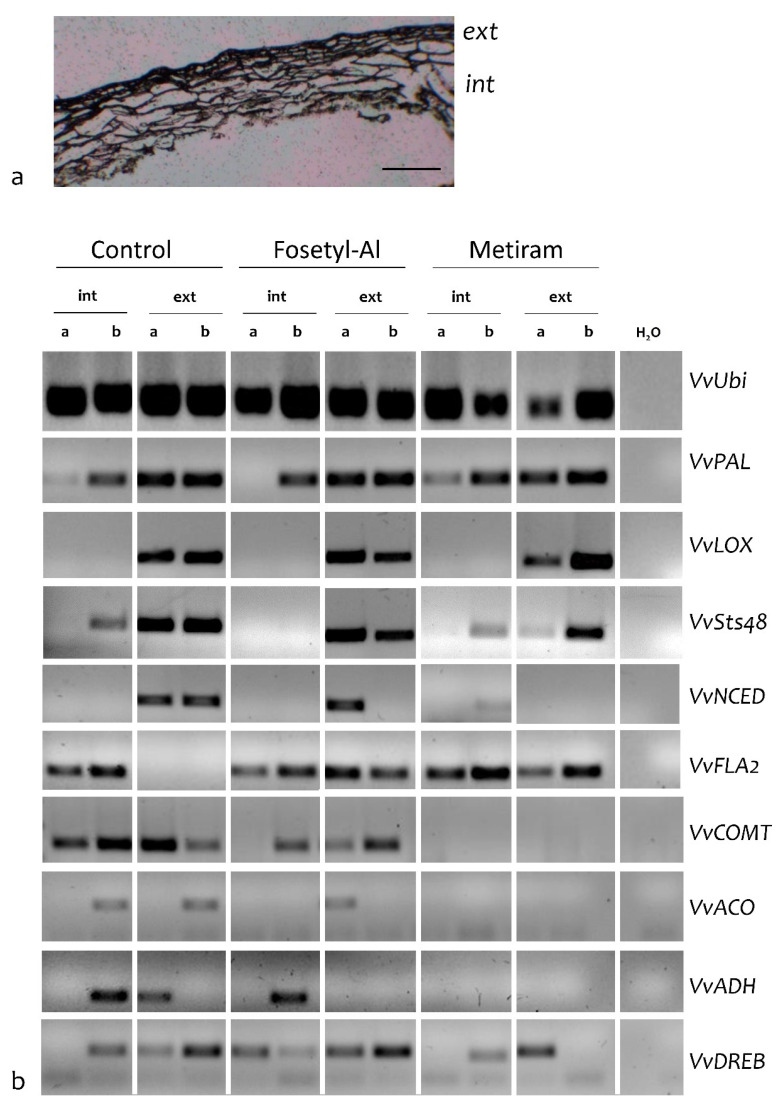
Gene expression analyses in microdissected samples. (**a**) Grapevine skin section where the external (Ext) and the internal (Int) layers, that were microdissected separately, are evident. Bar = 200 μm. (**b**) One-step RT-PCR analysis of microdissected cells. An amplified fragment of the expected size is present in all the samples using *VvUBI* as housekeeping gene. Using specific primers for selected genes significantly regulated in the RNAseq experiments, a fragment of the expected size is differentially present among the considered samples. For each treatment (CTRL, Fos-Al, Met), two biological replicates (**a**,**b**) for each cell-type population (int, ext) are shown.

Laser microdissection (LMD) technology was successfully applied to dissect two different cell-type populations distinguishable by an optical microscope, that is, (i) the external epidermis (called “ext”), characterized by regular small cells with moderately thick walls and (ii) the hypodermal layers (called “int”), formed by layers of cells increasing in size in the inner layers (Figure 3a). Based on the transcriptomic data of the whole berries and on the influences of the treatments on berry skin hardness and thickness previously reported [22], LMD experiments were performed on skins collected from berries of CTRL, Fos-Al and Met treated plants, mostly focusing on genes potentially involved in defence and in increasing penetration resistance in the berry cells. RNA isolated from laser microdissected cells was subjected to one-step RT-PCR. Transcripts corresponding to the following genes, previously considered for the RT-qPCR (Table S8 and Figure 2) were measured: a phenylalanine lyase (*VvPAL*), two stilbene synthases (*VvSTS16*, *VvSTS48*), a lipoxygenase (*VvLOX*), *VvNCED3*, an alcohol dehydrogenase (*VvADH*), a 1-aminocyclopropane-1-carboxylate oxidase 2 (*VvACO*). In addition, genes coding for a fasciclin-like arabinogalactan protein (VIT_12s0057g01020, *VvFLA2*), an O-methyltransferase putatively involved in monolignol biosynthesis (VIT_15s0048g02490; *VvCOMT*) and were analysed.

In RT-PCR experiments on microdissected samples, an amplified fragment of the expected size was observed in all cell types tested using specific primers for the housekeeping gene *VvUBI* (Figure 3b). Absence of an amplified product in RT minus reactions excluded genomic DNA contamination. In contrast to housekeeping genes, transcripts corresponding to considered genes presented different patterns of expression among treatments and cell-type populations. In detail, genes potentially involved in defence responses were exclusively (*VvLOX*) or mainly (*VvSTS48* and *VvPAL*) expressed in ext cell-type populations collected from CTRL and treated plants. The trend observed for *VvCOMT* in skin cell-type populations (absence in Met and presence in the Fos-Al) is of particularly interest due to the opposite expression revealed by RNAseq (Table 1 and Table S6). This gene appeared to be significantly upregulated in Met treated berries but not in those treated with Fos-Al. *VvNCED3*, which is involved in ABA biosynthesis, is also mainly expressed in ext samples in both CTRL and Fos-Al treated plants. The fact that in some cases there is not consistence between the two biological replicates, for example, for *VvNCED3* in Fos-Al, is probably related to the high variability observed in the samples. The same variability was observed in the RNA-seq experiment, with a limited number of DEGs identified among the treatments (Tables S5 and S6) and in the microbial and chemical-physical grape characteristics analysed in the same experiment [22]. Transcripts corresponding to *VvDREB*, coding for a dehydration responsive element binding, is mainly found in the skin cell-type populations in berries from Fos-Al treated plants, while they are observed only in some replicates and cell-types in the other treatments (CTRL and Met). *VvACO* and *VvADH* primers only led to a signal in one of the biological replicate in berries from CTRL and Fos-Al (in both int and ext samples) and did not lead to an amplification signal in samples from Met, suggesting that the skin is not the main site of expression for these genes. The same observation is valid also for *VvPIP2;2* whose transcripts have been found only in CTRL, although not in all the two biological replicates and for *VvXET* in one int replicate from Fos-Al (Figure S3).

## 3. Discussion

Currently, the factors leading to changes in berry features after treatments with antifungal treatments remain poorly studied. Previous works led to a comprehensive representation of gene expression dynamics in grapevine berries basing on transcriptome analysis [10,31,32,33] and genes expressed in skin and pulp were already identified [31,34] as well as those involved in the induction of the berry shrivel ripening physiological disorder in grapevine [28]. Additionally, Dal Santo et al. [6] have revealed candidate genes potentially responsible for the phenotypic plasticity of grapevine. Haile et al. [35] highlighted the cross talk between the plant and an important necrotrophic fungal pathogen in vineyards (i.e., *Botrytis cinerea*) by an integrated transcriptomic and metabolic analysis of the host and the pathogen. Here, the main purpose of the work was to deepen the long-lasting molecular defense priming features of different antifungal treatments in grapevine analyzing berry at harvest at the end of season. RNA-sequencing analysis was performed on berry samples collected from plants differently treated (Lam, EOW, resistance inducers, that is, Fos-Al and K-Pho in combination with sulphur and copper) in comparison with control untreated plants (CTRL) and Met, a standard chemical fungicide. Additionally, basing on sequencing and real-time PCR data, a laser microdissection experiment has been performed to verify the expression of selected genes on specific cell-type populations (epidermis and hypodermal layers) present in the skin.

About the transcriptomic data, it is worth noting that EOW led to expression changes of only three genes that resulted to be downregulated. This agrees with the data of infection levels in the field that showed the lowest powdery mildew incidence reduction as well as the lowest powdery mildew severity reduction with respect to other treatments [22]. For this reason, this treatment was not considered in the subsequent analyses on RNA-sequencing data.

All treatments modulated the berry transcriptome in a very limited way due in part to the great biological variability of the samples. This is foreseeable as the experiment was conducted in a commercial vineyard, even if the experiment was carefully carried out using randomized blocks to minimize the influences of the different microclimatic conditions in different areas of the vineyard, of the differences in the soil or in the phenological phases of the berry development. In addition, only asymptomatic berries were sampled in all theses to avoid influences due to differences in fungal attacks. The consistency of the berries at harvest in the various treatments was confirmed by the yield and quality data. In a previous work using the same samples, Rantsiou et al. [22] showed that the yield and vigor of vines as well as the primary and secondary metabolites produced in the berries were not influenced by the different treatments. Consequently, the molecular differences observed among the treatments are attributable to a systemic response induced in plants by these compounds. Additionally, a different disease incidence and severity was reported for the considered treatments [22]. The fact that they had only partial effect on transcriptional modulation at the end of season, on the grape quality and on microbial communities both in berry [22] and in leaf [18] suggests a specific action against pathogens and a low persistence over time of the molecular responses in plant several weeks after the treatments. Thus, our results offer novel information on long-term responses in a vineyard adopting defense protocols that can be used commercially.

### 3.1. A snapshot on the Berry Transcriptome

Overall, transcriptomic results confirm the different impacts of the several tested compounds [22]. Looking at the different treatments, it is worth noting that Lam led to the upregulation of only one gene coding for *VvNCED3* (VIT_19s0093g00550). Nine-cis-epoxycarotenoid dioxygenases (*NCEDs*) are encoded by multigene families and the expression of specific family members is deeply regulated in response to stress or developmental signals, contributing to ABA synthesis in different conditions [36]. In addition, ABA plays a relevant role in response to biotic stress by modulating the plant’s defenses against pathogens by interfering with other hormones and ROS accumulation [37,38]. Overexpression of a *NCED* in *V. vinefera* transgenic plants induced the production of jasmonic acid (JA) and accumulation of JA biosynthesis-related genes [39]. *VvNCED3* was previously reported as one of the three ABA-related transcripts significantly modulated in berries subjected to water deficit [7]. It was the only gene systemically upregulated in all the four treatments considered in transcriptomic analysis, suggesting a its relevant role in the triple interaction among antifungal compounds, berries at harvest and fungal pathogens and an involvement in the reduction of damage caused by fungal pathogens and environmental stresses on plants treated with antifungal compounds. Among the significantly upregulated genes in three conditions (Fos-Al, K-Pho, Met) with a putative role in defense and in increasing penetration resistance in the plant cells, a gene homolog with an aldehyde oxidase GLOX (i.e., glyoxylate oxidase) was detected. This enzyme, which is a copper-containing enzyme, was already reported as implicated in grapevine defense mechanisms in *Vitis pseudoreticulata* [40]. Another intriguing result is the upregulation of an omega-hydroxypalmitate O-feruloyl transferase homolog. This is an enzyme responsible for synthesizing suberin aromatics [41] and the increase in its transcripts might suggest again a reinforcement of the barrier against pathogens.

Among the genes specifically upregulated in only one treatment, it is worth noting the upregulation of a mannitol dehydrogenase in Fos-Al treated plants. This enzyme was reported to be secreted by the plants in response to pathogen attack as a defense against mannitol-secreting fungal pathogens and its upregulation was already proposed as a strategy for protecting plants against fungal pathogens that secrete mannitol as part of the infection process [42].

### 3.2. DEGs Associated to Secondary Metabolism and Cell-Wall Modifications

Despite that significantly differences in berry skin phenolic compositions have not been found among the several treatments [22], it is worth noting the downregulation of almost all the DEGs involved in response to stress and secondary metabolite categories, with a few exceptions in berries treated by Met. Stilbenes represent the most important class of phytoalexins in *Vitis* and accumulate in response to several environmental stress factors, including pathogen attack [43]. The biosynthetic pathway that leads to the stilbene production is a side branch of the general phenylpropanoid pathway and the key enzymes in the biosynthesis of these compounds are the stilbene synthases (STSs) that compete for the same substrates (p-coumaroyl-CoA and cinnamoyl-CoA) with chalcone synthases (CHSs), the key enzymes in the biosynthesis of flavonoids. In our work, several stilbene synthase and phenylalanine lyase genes but also a chalcone synthase gene, appeared to be downregulated among Fos-Al, K-Pho and Met treatments with respect to the berries from the CTRL plants. The same expression behavior observed here confirms that PAL genes are probably co-regulated with STSs during the biosynthesis of stilbenes, as previously reported [44]. Upregulation of STS genes and other plant defense genes was considered to contribute to the constitutive defense against pathogens during the development of grape berry and leaf [10] and references therein. Considering that our data are compared to infected control plants, it is possible to hypothesize that the downregulation of these genes is related to less incidence of powdery mildew infection, probably related also to a higher skin thickness with respect to CTRL [22]. An outer layer of hydrophobic cuticle waxes, usually considered as part of the skin, acts as the primary protective barrier against environmental stress [45]. Interestingly, a wax synthase gene (VIT_12s0028g03480), previously found as slightly upregulated during ripening [30], is weakly but significantly upregulated in berries from the three most significant considered treatments (Fos-Al, K-Pho and Met). Despite these observations on the skin features, we cannot exclude also that the observed patterns for these genes might have a time-dependent regulation, considering that berries were sampled at harvest. For example, *VvSTS16* and *VvSTS48* have shown in berries of ‘Nebbiolo’ a highly variable expression depending on genotype (clone), developmental stages, environment and vintage [10]. Further studies are needed to decipher the possible regulatory mechanisms involved in the downregulation of several STS genes in berries from antifungal treated plants.

Unexpectedly, only a few cell wall related genes appeared to be regulated and among them, a gene coding for a putative xyloglucan endotransglucosylase/hydrolase (VIT_01s0150g00460) was the highest upregulated gene in Fos-Al (fold = 2.6). The importance in xyloglucan metabolism in cell wall rearrangement during ‘Corvina’ berry ripening was demonstrated [30] as well as in ‘Cabernet Sauvignon’ and ‘Crimson Seedless’ berries [46]. The same XET gene was highly overexpressed in ‘Nebbiolo’ berries at véraison [10] and marginally induced during ripening in ‘Corvina’ berries [6]. XET are generally induced by sugars [2] and ABA [47] and consequently the overexpression of *VvNCED3* may be linked to the upregulation of *VvXET*. By contrast, two genes related to cellulose metabolism, that is, VIT_01s0137g00430 and VIT_02s0025g01780 coding for a putative cellulase and cellulase synthase, respectively and four genes involved in pectin modifications, that is, three pectinesterases VIT_15s0048g00510, VIT_15s0048g00500, VIT_16s0022g00700) and a polygalacturonase (VIT_08s0007g08330) were regulated by at least one treatment. The dynamic expression profiles of cell wall-modifying enzymes and corresponding changes in cell wall composition reveal that pectin modification was reported to be one of the major responsible for the loss of firmness in ripening and withering fruits [30,48]. Again, a pectinesterase gene (VIT_15s0048g00500) was already found to be strongly upregulated during both ripening [10,30] and withering [48]. The fact that these genes, all known to exert a role in berry development [34,49] and previously observed as upregulated during ripening in ‘Nebbiolo’ [10], are all downregulated by the treatments suggest a difference in berry textural properties related to cell disruption during ripening between treated- and control plants. This is particularly important since these changes may be associated with the release of aroma compounds due to the interaction among enzymes and substrates previously compartmentalized.

### 3.3. Cell-Specificity in Two Different Cell-Type Populations in Grape Berry Skin

Grape berries are formed by the mesocarp (pulp), characterized by cells with thin cell walls and the exocarp (skin), containing thick-walled epidermal and hypodermal cells [30]. The skin cell wall structure and composition are features with a great importance for the extractability of phenolics and other compounds during wine production as well as for aspects correlated to skin hardness that could influence food quality of table grapes [22,30,48]. The effect of antifungal treatments on berry skin hardness and thickness have been previously evaluated [22]. Despite significant differences for skin hardness were not found for any treatment when compared to CTRL, Lam showed the highest values of skin hardness. Furthermore, all treatments increased the skin thickness of berries in comparison to CTRL and particularly Fos-Al showed a significant increase of this parameter [22]. Even small variations in skin hardness and thickness can influence the extractability of phenolic substances during the maceration, an aspect very important for ‘Nebbiolo’ is the richness in 3’-hydroxylated anthocyanins [50]. Indeed, the wine produced form the berries of ‘Nebbiolo’ subjected to antifungal treatments showed higher quantities of anthocyanins in comparison to wine produced from CTRL with a positive impact on the color intensity of wine [22].

Several studies have already identified differences between the skin and pulp cell walls, also reflecting differences among cultivars [30,48]. A different dynamic in cell wall composition in the external and internal skin tissues during ripening and withering of ‘Corvina’ berries has been already demonstrated [30,48]. In this work, cell-specific expression in the external and internal berry skin cell-type populations was followed by using an LMD approach combined with one-step RT-PCR, focusing on fourteen DEGs involved in different processes. As a first result, a protocol to apply this technology to collect separately two different cell-type populations grape berry skin was obtained. LMD technology was already used to dissect the complexity in citrus and tomato fruits [51,52]. In grapevine, LMD was applied to analyze the transcriptional changes in stomata cells and surrounding areas of grapevine leaves at early stages of *Plasmopora viticola* infection [53], to identify the site-specific gene responses in grapevine leaf phloem infected by stolbur [54] as well as to verify the involvement of vessel-associated cells in embolism recovery in leaf petiole [55]. Looking at the expression of considered genes in the two cell-types, it is worth noting the presence of the expression of three genes potentially involved in the defense response (*VvLOX*, *VvPAL* and *VvSTS48*) in the external skin layer. Interestingly, the predominant presence of genes involved in defense responses in the epidermis was already reported in citrus [51]. The compounds used in this work act on the surface of the leaves and berries where they are sprayed, therefore the external cells, that are the first barrier of protection against the fungal pathogens, seem to be the main factors responsible for the responses of the berries to the treatments. The absence of transcript corresponding to *VvCOMT* in berries collected from Met is worth to be noted, considering its upregulation in berries treated with Met and K-Pho in transcriptomic data. This result suggests that the site-specific regulation might be dependent on the treatment. Results also confirmed the great variability within each treatment. Transcripts corresponding to specific genes (e.g., *VvNCED3*) were in fact found only in one biological replicate, at least for some treatments. This is not surprisingly considering that also several parameters previously measured were not significantly regulated due to the great variability among replicates expected for environmental samples [22].

To conclude, it has been shown that all the five considered antifungal treatments do not strongly systemically affect the berry transcriptome profile at the end of season. In detail, the conventional one (Met) led to a higher number of DEGs with respect to the other treatments, suggesting that this one could have a major impact on berry physiology, with respect to the other antifungal treatments, that should be further investigated. The greater overlap of molecular responses among Met, K-Pho and Fos-Al may suggest that fosetyl-Al and potassium phosphonate might be potentially useful to compensate for the reduction of copper in the future, although further pathological, physiological and molecular experiments will be needed. Additionally, results demonstrate that the coupling of LMD with targeted gene expression analysis is a powerful tool to identify cell-type-specific transcripts, providing new insights into cell-specific gene expression aspects and leading to a better understanding of the specialized contribution of each tissue to skin/fruit physiology. Genes differentially expressed in the two-cell type populations forming the berry skin was in fact observed, suggesting a different function for the two-cell type populations. Some candidate genes correlated to defense responses appeared in fact to be more or exclusively expressed in the external cell-type population.

## 4. Materials and Methods

### 4.1. Field Trials

The field trial was carried out on wine grape ‘Nebbiolo’ (*Vitis vinifera* L.) in a vineyard located in Piobesi D’Alba (North-West Italy) (GPS: 44.731760, 7.988324, hill area) during 2017. The climatic data of the vineyard were collected in 2017 (Figure S1). Vines were planted in parallel and contiguous rows, vertically trained, Guyot pruned and all grafted on the same rootstock (Kober 5BB). The distance between vines was 0.90 × 2.5 m. Vineyard management was uniform in the experimental site and in compliance with best agricultural practices of the growing location. The experimental scheme included four randomized blocks per treatment, each containing eight plants as described previously in detail [22]. Experimental tested products were applied with a hand-pulled 2-stroke engine sprayer at a pressure of 15 bar distributing 400–600 L/ha. The plants were treated until near run-off. The commercial antifungal products were used according to the manufacturer’s information. The products were compared with an untreated control (CTRL). The commercial formulations applied were: fosetyl-Al (80% a.i., Aliette, Bayer CropScience, Monheim am Rhein, Germany), potassium phosphonate (755 g/L a.i., Century, BASF Agro, Cesano Maderno, Italy), laminarin (Vacciplant 45 g/L a.i., Arysta Lifescience, Agrate Brianza, Italy), metiram (70% a.i., Polyram, BASF Agro), electrolyzed water (sodium hypoclorate, EVA System^®^, De Nora S.p.A., Milan, Italy). These commercial products were applied in combination with sulphur and copper as indicated in Table S1. The efficacy of the different treatments evaluated as percentage of infected grape clusters and berries was reported in Rantsiou et al. [22].

### 4.2. Grape Sampling

Berry sampling was performed in the vineyard at harvest in 2017 for RNAseq and an additional sampling was done in 2018 for laser microdissection experiment. Grape berries were sampled from the upper, middle and distal part of the bunch, alternatively from the shaded and from the exposed side of the cluster and from each side of the row. Only asymptomatic berries without symptoms of fungal attacks were collected for each treatment and block with the pedicel attached were randomly selected and collected into sampling bags. The samples were transported in the laboratory and processed immediately for laser microdissection or kept in −80 °C for transcriptomics.

### 4.3. RNA Extraction and Illumina Sequencing

For the RNASeq experiment, 10 deseeded berries, from at least three different grape clusters, for each treatment-block combination (3 replicates for 6 treatments = 18 samples) were chilled in liquid N2 and RNA was extracted using the “pine tree- method” [56] with the addition of 2% PVPP to the extraction buffer. RNA quality and quantity controls have been performed using the Agilent 2100 Bioanalyzer. Ten micrograms of each RNA sample (RIN ≥8) were sent to (Macrogen Inc., Seoul, South Korea) where the libraries were produced and sequenced using an Illumina Genome Analyzer (Solexa). The eighteen libraries were indexed and single-end multiplexed sequencing was performed using 100-bp length reads. The reads obtained from Illumina HiSeq were processed using CASAVA pipeline version 1.8.2. (Illumina Inc., San Diego, CA, USA) and further checked for sequence quality with the fastQC application (ver. 0.10.1).

### 4.4. RNA-Seq Analysis

All the 18 libraries were QA pre-processed prior analyses with RQCFilter script, implemented in the BBTOOLS suite v.37.74 (https://sourceforge.net/projects/bbmap/, last accessed 2020-02-04). The automated pipeline performed adapter-trimming, quality-trimming and filtering (min. qual. score = Q25, min. frag. len. = 49), contaminant removal (including common bacterial species, human, cat, dog and mouse sequences) and ribosomal, chloroplast and mitochondrial sequences from Illumina reads. Reads mapping were performed with STAR v.2.7.3a (cit.) using the *V. vinifera* genome assembly 12X, INSDC Assembly GCA_000003745.2 (Nov 2011) and annotation as reference (release 40, 2018-07-17), available at Ensembl Plants database (https://plants.ensembl.org/Vitis_vinifera/Info/Index, last accessed 2020-02-04). The resulting 18 BAM files, with the reads aligned on the reference, were loaded into the R project statistical environment (R Core Team, 2013) for the detection of DEGs. The function featureCounts from the RSUBREAD v.1.34.7 package [57] was used to assign mapped reads to genomic features, namely genes, counting only read pairs with both ends mapped and filtering alignments showing the minimum mapping quality score set to 30. DEG analysis was conducted using the DESeq2 v1.24.0 package [58] setting the design formula to take into account for the 5 formulates against the control (design = ~ formulate). For DEG analyses, the untreated control (CTRL) was set as reference level. Weakly expressed genes were filtered by means of the function HTSFilter, from the package HTSFILTER v.1.24.0 [59], which implements a data-based filtering procedure based on the calculation of a similarity Jaccard index among biological replicates for read counts arising from replicated RNA-seq data and using the “Trimmed Mean of M-values” (TMM) as normalization method to correct for differences in library sizes. Due to the usage of this filtering method, the independent Filtering option in the results function, used to extract results from a DESeq2 analysis, was set to FALSE. Statistically significant genes were selected basing on the adjusted *p*-value, with the threshold set to 0.05. Up or Down regulated genes were recognized by means of their log2 Fold Change values and exported in excel files for downstream analyses. Gene Ontology (GO) enrichment analysis was carried out using the BiNGO 3.0 plug-in tool in Cytoscape v. 3.2 and over-represented Plant GO categories were identified using a hypergeometric test with a significance threshold of 0.05. Selected proteins were further analyzed using Blastp and Interpro tools, mainly when grapevine annotation was not satisfactory. The dataset (raw data) that supports the findings of this study is available in the NCBI Sequence Read Archive (SRA-NCBI) at https://www.ncbi.nlm.nih.gov/sra under BioProject accession number PRJNA607172 and BioSample accession numbers SAMN14123560-SAMN14123577.

### 4.5. Real-Time Quantitative PCR Analysis Validation

For quantitative PCR analyses (RT-qPCR), total RNA was extracted using the Spectrum™ Plant Total RNA extraction kit (Sigma Aldrich, Saint Louis, MO, USA) starting from 100–200 mg of a pool of deseeded berries and total RNA yield was determined using a NanoDrop spectrophotometer (Thermo Fisher Scientific, Waltham, MA, USA). Genomic DNA was removed using the Turbo DNA-free™ reagent (Thermo Fisher), according to the manufacturer’s instructions. Before cDNA synthesis, RNA was subjected to a reverse transcription-PCR reaction (RT-PCR) to exclude DNA contamination using the One-Step RT-PCR kit (Qiagen, Hilden, Germany). SuperScriptII Reverse Transcriptase (Thermo Fisher) was used to synthesize cDNA starting from 500 ng of total RNA for each sample, following the manufacturer’s instructions. Table S9 reports the primers for RT-qPCR. Quantitative PCR was performed with the Rotor-Gene Q (Qiagen) apparatus. Reactions were carried out in a final volume of 15 μL with 7.5 μL of Rotor-GeneTM SYBR^®^ Green Master Mix, 5.5 μL of a mix of forward and reverse primers (prepared by adding 16 μL of each primer at 10 μM stock concentration to 168 μL of water) and 2 μL of cDNA (diluted 1:5). RT-qPCR cycling program consisted of 10 min/95 °C holding step followed by 40 cycles of two steps (15 s/95 °C and 1 min/60 °C). Each amplification was followed by melting curve analysis (60–94 °C) with a heating rate of 0.5 °C every 10 s. All reactions were performed with two technical replicates and only Ct values with a standard deviation that did not exceed 0.3 were considered. The comparative threshold cycle method was used to calculate relative expression levels using the grapevine *VvUbi* and *VvAct* as reference genes. Statistical analyses were performed through one-way ANOVA and Tukey’s post-hoc test, using a probability level of *p* ≤ 0.05. All statistical elaborations were performed using the PAST statistical package (version 2.16).

### 4.6. Laser Microdissection

#### 4.6.1. Tissue Preparation for LMD

For paraffin embedding, berry grape skins were isolated with a razor blade in fixative and fixed in freshly prepared Farmer’s fixative (absolute ethanol/glacial acetic acid 3:1) at 4 °C overnight. Samples were subsequently dehydrated in a graded series of ethanol (70%, 90% in sterile water, 100% twice) followed by two incubations in Neoclear (Merck), with each step on ice for 30 min and embedded in paraffin. Neoclear was then gradually replaced with paraffin (Paraplast Plus) and samples were embedded in paraffin in Petri dishes. Sections of 8 μm thickness were cut using a rotatory microtome, placed and stretched out on Leica RNase-free PEN foil slides with dH_2_O (filtered with a 0.2 μm filter). Sections were then dried on a 40 °C warming plate, stored at 4 °C and used within 2 days.

#### 4.6.2. Collection of Specific Cell-Type at Laser Microdissection

A Leica LMD 6500 Laser microdissection system (Leica Microsystems, Wetzlar, Germany) was used to isolate the different cell-types from the prepared tissue sections [60]. Just before use, the slides with the sections were de-paraffinized with Neoclear^©^ (Merck, Darmstadt, Germany) for 8–10 min, rinsed in 100% ethanol for one minute and then air-dried. De-paraffinized slides were placed face-down on the microscope and two different cell-type populations (epidermal and hypodermal skin layers) were selected from the berries, microdissected and collected separately. Approximately, 150-100 skin fragments for each cell-type were collected for each of the two independent biological replicates and the pools were brought to a final volume of 50 μL with Pico Pure extraction buffer and processed for RNA extraction.

#### 4.6.3. RNA Extraction and RT-PCR from Microdissected Cells

RNA was extracted using the Pico Pure kit (Life Technologies), without DNase treatment in the kit column. The RNA was eluted in 21 μL of Elution buffer and treated with RNase-free DNAse (TURBO™DNase, Ambion, Thermo Fisher), according to the manufacturer’s instructions. A One-Step RT-PCR kit (Qiagen) was used for the RT-PCR experiments on RNA extracted from the different LMD samples. All RNA samples were checked for DNA contamination through RT-PCR analyses with specific primers for VvUbi (Table S9), without a previous retro-transcription step (RT-). RT-PCR assays on targeted genes (Table S9) were carried out using a one-step protocol. The samples were incubated for 30 min at 50 °C, followed by 15 min of incubation at 95 °C. Amplification reactions were run for 40 cycles at 94 °C for 30 s, 60 °C for 30 s and 72 °C for 40 s. The RT-PCR experiments were conducted on two different biological replicates. The PCR products were visualized by agarose gel electrophoresis.

## Figures and Tables

**Table 1:** List of the unique DEGs among the top 10 from each treatment.

Gene ID	Putative Function *	Fold Change in Each Treatment		
		Fos-Al	K-Pho	Met
VIT_00s0207g00010	ω-hydroxypalmitate O-feruloyl transferase	1.6	1.7	1.8
VIT_02s0025g02600	Aldehyde oxidase GLOX	1.8	1.7	1.7
VIT_07s0031g02200	Auxin efflux carrier-like protein (PIN-LIKE)	0.7	0.8	0.7
VIT_07s0031g03220	Patellin protein	1.2	1.1	1.2
VIT_12s0028g03480	O-acyltransferase WSD1	1.7	1.9	1.9
VIT_19s0093g00550	9-cis-epoxycarotenoid dioxygenase (*VvNCED*)	1.0	0.9	1.0
VIT_01s0150g00460	Probable xyloglucan endotransglucosylase/hydrolase (*VvXET*)	2.6		2.2
VIT_08s0007g01900	Proton-dependent oligopeptide transport (POT) family protein	1.6		1.5
VIT_13s0067g00110	Cytochrome P450 family	2.0		2.0
VIT_13s0074g00390	Cytochrome P450 family	1.4		1.3
VIT_15s0048g02480	O-methyltransferase		2.5	2.5
VIT_15s0048g02490	O-methyltransferase (COMT type)		2.5	2.5
VIT_17s0000g08070	Aldehyde dehydrogenase family			0.6
VIT_18s0001g03910	Nitrate reductase 2 (NR2)			0.9
VIT_18s0001g04470	TGACG MOTIF-binding factor 4 (transcription factor TGA4)			0.6
VIT_18s0001g08100	Zinc finger family protein			0.9
VIT_18s0001g11600	Protein JINGUBANG			1.1
VIT_18s0001g12660	TUBBY like protein			1.3
VIT_18s0001g15330	Bidirectional sugar transporter SWEET (Nodulin MtN3)			1.3
VIT_18s0072g00970	DegP protease			0.8
VIT_19s0015g01270	Proteasome activator subunit 4			0.8
VIT_18s0001g14910	Mannitol dehydrogenase	1.7		
VIT_18s0001g08090	Auxin-responsive protein		0.7	
VIT_19s0085g00920	Organic cation transport protein OCT2		0.9	

* Putative function is based on the grapevine genome annotation and on the best Blastp (ref-seq).

## Supplementary Materials

Supplementary materials can be found at https://www.mdpi.com/1422-0067/21/17/6067/s1.

## Abbreviations

Fos-Al	Fosetyl-Al
K-Pho	potassium phosphonate
Lam	Laminarin
LMD	Laser microdissection
Met	Metiram
