# Supplementary material for: Long-Term Impact of Chemical and Alternative Fungicides Applied to Grapevine cv Nebbiolo on Berry Transcriptome
Source: Int J Mol Sci. 2020 Aug 23;21(17):6067. doi: 10.3390/ijms21176067 (PMC7504522; doi:10.3390/ijms21176067)
Supplement: Supplementary file 1 [file ijms-21-06067-s001.zip › supplementary Figure.pdf]

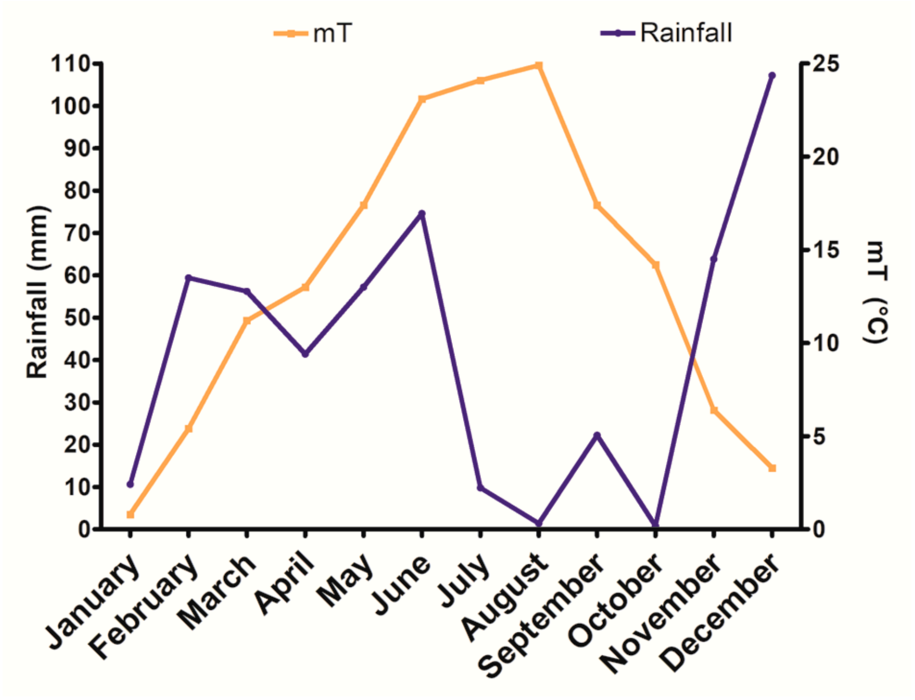

Figure S1. Climatic data.

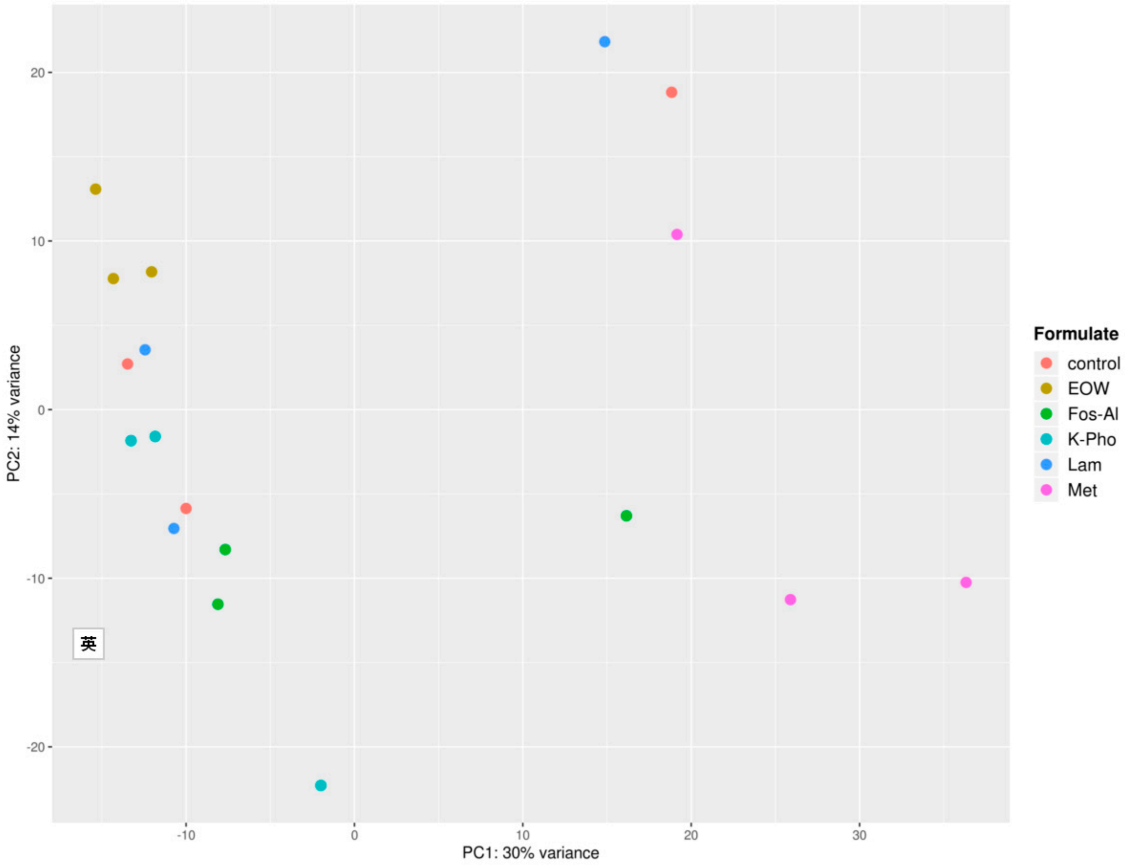

Figure S2. Sample Principal Component Analysis (PCA) plot for transformed data (log2 normalized counts), visualizing the overall effect of experimental covariates and batch effects in the different replicates of the several treatments: control, Fos-Al (Aliette), K-Pho (Century), EOW (Electrolyzed water), Met (Polyram), Lam (Vacciplant).

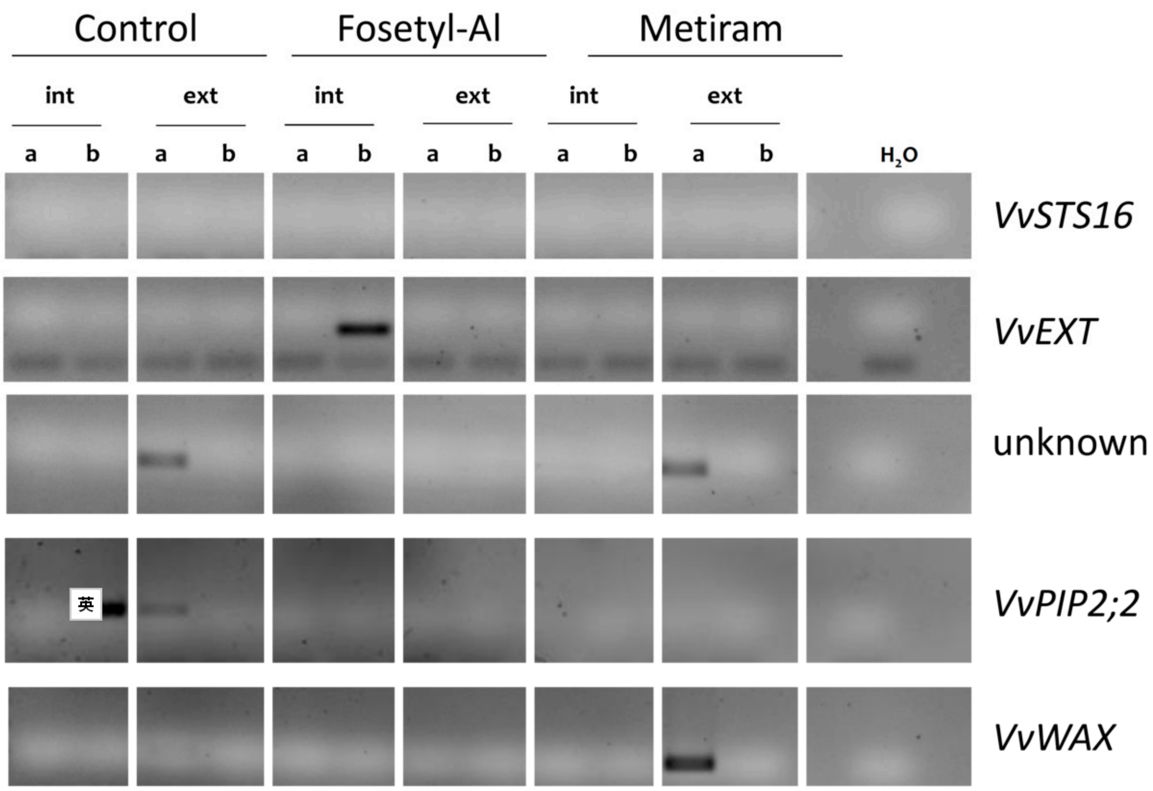

Figure S3. One-step RT-PCR analysis of microdissected cells using specific primers for *VvST16*, *VvEXT*, an unknown protein, *VvPIP2;2* and *VvWAX*.
